# The Use of Radiosynovectomy Using Yttrium-90 as an Adjunct to Mechanical Synovectomy for Pigmented Villonodular Synovitis: How Effective Is This?

**DOI:** 10.7759/cureus.69520

**Published:** 2024-09-16

**Authors:** Mina Al-Janabi, Ifeanyi K Onubogu, Karl F Almqvist, Hesham Al-Khateeb, Taiceer Abdulwahab, Mazin A Janabi

**Affiliations:** 1 Radiology, Princess Alexandra Hospital, Harlow, GBR; 2 Trauma and Orthopaedics, Norfolk and Norwich University Hospital, Norwich, GBR; 3 Trauma and Orthopaedics, Orthocure, Dubai, ARE; 4 Trauma and Orthopaedics, Clemenceau Medical Center Hospital, Dubai, ARE; 5 Trauma and Orthopaedics, Mediclinic City Hospital, Dubai, ARE; 6 Nuclear Medicine, Mediclinic City Hospital, Dubai, ARE

**Keywords:** arthroscopy, debridement, knee, pigmented villonodular synovitis, pvns, radiosynovectomy

## Abstract

Introduction

Pigmented villonodular synovitis (PVNS) or giant cell tumour of the tendon sheath (GCTT) is a rare disorder involving the proliferation of the synovium in any joint; however, the knee, hip, and ankle joints are most commonly targeted.

The aim of this study is to follow the short-term outcomes of the treatment of patients with PVNS of the knee managed by intra-articular injection of yttrium-90 or radiosynovectomy (RS) alone and a combination of RS and arthroscopic/open debulking synovectomy.

Methods

A cohort of eight knees from six patients was included in this study, including three knees treated with combined arthroscopic synovectomy and RS, and five knees treated with RS alone. Patients were asked to complete the Knee Injury and Osteoarthritis Outcome Score (KOOS) questionnaire before attending their three-month follow-up appointment. Their answers were collated and scores were calculated using the designated specific equation.

Results

Our results demonstrate that although patients in the combined arthroscopic synovectomy and RS arm possess on average higher pain scores and symptoms than those who underwent RS alone, the combined therapy had higher scores in activities of daily living (ADLs), sports and recreation (Sport-Rec), quality of life (QoL), and overall KOOS scores.

Conclusion

Neoadjuvant arthroscopic mechanical debridement is a safe and quick procedure with minimal side effects, with improved overall KOOS scores.

## Introduction

Pigmented villonodular synovitis (PVNS) or giant cell tumour of the tendon sheath (GCTT) is a rare disorder involving the proliferation of the synovium in joints; however, the knee, hip, and ankle joints are most commonly affected [1]. It is usually mono-articular although it can rarely target multiple joints, with the knee most commonly targeted. PVNS characteristically affects younger individuals in the third to fourth decade of life [2]. The condition can be categorised into local and diffuse forms, with the former affecting the tendons of a joint or limited to part of a joint [3]. Furthermore, in its localised form, PVNS results in pedunculate nodules. The diffuse variant presents more aggressively, affecting the entire joint, and may even extend beyond the joint [4]. Macroscopically there is proliferation of the synovial membrane with haemosiderin deposition. Characteristically, the microscopic disease process demonstrates hyper-proliferation of stromal cells and fibroblasts with the presence of lipid-laden macrophages and multinucleated giant cells [3]. If left untreated, PVNS can culminate in the progressive destruction of joints, resulting in a loss of joint function. This ultimately predisposes patients to develop secondary osteoarthritis [4].

Patients typically present with mono-articular swelling, pain, and loss of function. The disease process is often gradual; however, in light of its rarity, PVNS can be difficult to identify and distinguish from other rheumatological conditions. Consequently, a delayed diagnosis may ensue, at which point evidence of joint destruction and functional deficit may already be present [5].

Diagnosis involves a thorough clinical assessment supplemented by other investigations. Clinical examination may reveal a swollen joint with an effusion. Initial radiographs may be normal or demonstrate a reduction in joint space, evidence of osteoarthritis, and, in severe cases, bony destruction. Synovial thickening can sometimes be seen on magnetic resonance imaging (MRI). Aspiration of affected joints may indicate an ongoing inflammatory process and is commonly extensively blood-stained. Synovial villi sediment may also be present in synovial fluid samples [6]. Ultimately, a definitive diagnosis is achieved with a tissue biopsy obtained from an arthroscopic or open procedure for histological assessment. Samples can reveal characteristic features of synovial hyperplasia [3].

The management of PVNS is primarily aimed at providing symptomatic relief, preventing further progression of the disease and ultimately preserving joint function. As the condition progresses and joint destruction ensues, the likelihood of arthroplasty increases, which is particularly undesirable in the young cohort of patients. Therefore, protecting the native joint is a priority. At present, there is no optimal or gold standard treatment for patients diagnosed with this condition; however, various treatment options have been described ranging from marginal excision for localised pathology to subtotal/total synovectomy for diffuse PVNS. This can be achieved via arthrotomy or arthroscopically with the latter being less invasive and enabling a quicker recovery and lower morbidity to the patient. In cases where there is an extensive diffuse pattern of PVNS, complete synovectomy is required [7]. Complete excision of the synovium may be unachievable via arthroscopy and access to the posterior synovium in particular can be difficult. As a consequence, arthroscopic synovectomy possesses a higher rate of disease recurrence particularly with diffuse PVNS and further surgery may be required. In addition, it can be challenging to achieve a total synovectomy without impairment of the articular surface, which itself may pose further problems [8]. In addition to surgical management, radiosynovectomy (RS) has also been described. This can be achieved using external beam radiotherapy and intra-articular yttrium-90 (90Y) injection into the knee. Specifically, RS involves the use of beta-emitting radioactive substances to irradiate the synovium of the intra-articular joint of the knee [7]. Both modalities mentioned have demonstrated effectiveness in lowering the rate of local disease recurrence [9]. Injection of 90Y has the potential to achieve a complete synovectomy without the added risk of joint damage associated with a mechanical synovectomy. The adverse effects of 90Y have been reported to be minimal, making it a desirable treatment option over surgical management.

The aim of this study is to follow the short-term outcomes of patients with PVNS of the knee, treated in a single unit by observing post-treatment responses and function with the use of a validated questionnaire.

This article was previously presented as a meeting abstract at the American College of Surgeons Annual Conference in October 2022 and the ISAKOS Congress in June 2023.

## Materials and methods

This retrospective case series aims to assess the short-term outcomes of treatment of PVNS in the knee joint. All patients with biopsy-confirmed PVNS presenting to Mediclinic City Hospital (MCH), UAE between January 2018 and January 2021 were included. The inclusion criteria involved patients over 18 years of age with histological evidence of diffuse PVNS of the knee who underwent arthroscopic debridement, followed by radiosynovectomy. Apart from previous arthroscopy or open debridement of the affected knee, no other prior procedures were permitted and patients with a history of previous radiosynovectomy of the joint were excluded. Patients with a known inflammatory/rheumatological disorder affecting the knee in question were also excluded from the study, in addition to those with a history of lower limb trauma and those with a severe congenital anomaly/deformity.

The objective of this study is to assess patients’ response and function following treatment. To evaluate this, we employed the Knee Injury and Osteoarthritis Outcome Score (KOOS) as our primary outcome measure. This is a validated score that asks the patient questions relating to their symptoms, including stiffness, pain, function/daily living, function, sports/recreational activities (Sport-Rec), and quality of life (QoL) [10]. Responses were gathered from patients at their three-month follow-up following treatment.

A cohort of eight knees from six patients was included, including three knees treated with combined arthroscopic synovectomy and radiosynovectomy, and five knees treated with radiosynovectomy alone. Two of the six patients underwent arthroscopic synovectomy followed by radiosynovectomy, with one patient undergoing the procedures bilaterally (a total of three knees combined arthroscopic synovectomy followed by radiosynovectomy) The remaining group of four patients were administered an intra-articular injection of 90Y only, with one patient receiving bilateral radiosynovectomy (total five knees undergoing radiosynovectomy alone).

For combined therapy, the time interval between arthroscopic synovectomy and radiosynovectomy was six weeks. Guidelines from the European Association of Nuclear Medicine were followed for the administration of 90Y [11].

Radiosynovectomy protocol

Yttrium-90 emits a beta particle with a maximum energy of 2.27 MeV, a mean energy of 0.935 MeV, and an average soft tissue range of 3.6 mm. The recommended activity for the knee joint is 185-222 Mbq (5-6 mCi). The physical half-life is 2.7 days. This is the optimum dosage for efficacy, with limited toxicity and a short half-life [11].

Our cohort did not possess any contraindications for radiosynovectomy, which include pregnancy, breast-feeding, ruptured Baker’s cyst, or local signs of infection. Patients were informed of the potential complications of treatment, such as haemarthrosis, infection, and extravasation, as well as the risk of exposure to beta-emitting radiation and potential consequent side effects including radiation necrosis (rare) and future malignancy.

Yttrium-90 diluted with 0.9% sodium chloride (NaCl) to a volume of 2 ml was injected into the supra patellar pouch. This was followed by 20 mg triamcinolone hexacetonide in combination with 1 ml 2% lidocaine-HCl to avoid local skin reaction due to spill upon withdrawal of the syringe. The knee was cycled several times to evenly distribute the fluid throughout the joint. The patient was then advised bed rest and immobilisation of the knee for 72 hours post-injection to reduce the transport of particles through the lymphatics to the regional lymph nodes [11].

No patients included in our study underwent repeated treatments in the same joint during the follow-up interval.

Patients from both groups were asked to complete the KOOS questionnaire following their respective procedures. All responses were collated on arrival at the three-month clinic follow up and scores were calculated using the designated specific equation. The average score from a subsection is multiplied by 100 with the subsequent answer divided by four (the maximum number of points that can be obtained per question). This figure is then subtracted from 100 with the resulting number representing the final KOOS score. A score of 100 implies that the patient is asymptomatic whereas a score of 0 indicates that the patient is experiencing severe symptoms affecting their QoL.

## Results

For the individual variables, data were entered using IBM SPSS for Windows version 25.0 (IBM Corp., Armonk, NY). The measure of tendency and dispersion were performed as descriptive. Shapiro-Wilk test was used to test the normality of continuous variables pain, symptoms, activities of daily living (ADL), Sport-Rec, and QoL.

To compare normally distributed variables, an independent t-test was used, otherwise, the Mann-Whitney test was employed. A P-value of less than 0.05 was considered significant in all statistical analyses. The Shapiro-Wilk test has demonstrated that the KOOS measurements of pain, symptoms, and ADLs are normally distributed while Sport-Rec and QoL are not normally distributed (Table 1).

**Table 1:** Test of normality of continuous variables. ADL: activities of daily living; Sport-Rec: sports and recreation; QoL: quality of life; df: degrees of freedom.

	Statistical value (Shapiro-Wilk test score)	df	P-value
Pain	0.932	8	0.534
Symptoms	0.898	8	0.28
ADL	0.912	8	0.368
Sport-Rec	0.8	8	0.028
QoL	0.798	8	0.028

Patients from the combined synovectomy cohort demonstrated higher average KOOS scores in all subcategories compared with patients who underwent radiosynovectomy alone. The average of all KOOS scores for the combined arm is also higher (75) than the radiosynovectomy arm (29) (Tables 2, 3).

**Table 2:** Knee Injury and Osteoarthritis Outcome Score (KOOS) following radiosynovectomy alone. ADL: activities of daily living; Sport-Rec: sports and recreation; QoL: quality of life.

	Pain KOOS score	Symptoms KOOS score	ADL KOOS score	Sports-Rec KOOS score	QoL KOOS score	Average KOOS score
Patient 1 knee 1	28	36	46	0	0	22
Patient 1 knee 2	28	36	46	0	0	22
Patient 2	53	32	77	0	0	32.4
Patient 3	58	79	85	35	6	52.6
Patient 4	17	21	18	0	25	16.2
Mean score	37	41	54	7	6	29

**Table 3:** Knee Injury and Osteoarthritis Outcome Score (KOOS) following combined arthroscopic & radiosynovectomy. ADL: activities of daily living; Sport-Rec: sports and recreation; QoL: quality of life.

	Pain KOOS score	Symptoms KOOS score	ADL KOOS score	Sports-Rec KOOS score	QOL KOOS score	Average KOOS score
Patient 1 knee 1	86	75	99	70	69	79.8
Patient 1 knee 2	100	86	100	80	88	90.8
Patient 2	58	57	76	60	25	55.2
Mean score	81	73	92	70	61	75

Statistical significance is noted for average KOOS scores in the subcategories relating to pain, Sports-Rec, and QOL (0.04, 0.017, and 0.031 respectively). No statistical significance is demonstrated in either cohort with regard to average KOOS scores for symptoms and ADLs (Table 4).

**Table 4:** Variables between arthroscopic synovectomy followed by radiosynovectomy vs. radiosynovectomy alone. KOOS: Knee Injury and Osteoarthritis Outcome Score; ADL: activities of daily living; Sport-Rec: sports and recreation; QoL: quality of life; SD: standard deviation.

Variables	Arthroscopic synovectomy followed by radiosynovectomy	Radiosynovectomy alone	P-value
	Mean KOOS score (SD)	Mean KOOS score (SD)	
Pain	81.33 (21.39)	36.8 (12.35)	0.04
Symptoms	72.67 (17.72)	40.8 (22.22)	0.07
ADL	91.67 (13.58)	54.4 (26.99)	0.072
Sport-Rec	70 (10)	7 (15)	0.017
QoL	60.67 (32.32)	6 (10)	0.031

## Discussion

Despite our small sample size, the KOOS measurements of pain, symptoms, and ADL are all normally distributed and could theoretically be extrapolated to larger sample sizes. Our results demonstrate that patients treated with combined arthroscopic synovectomy and radiosynovectomy possess on average higher pain scores and symptoms than those who underwent radiosynovectomy alone; however, those treated with combined therapy had higher scores in ADLs, Sport-Rec, QoL, and overall KOOS scores.

With reference to previous studies, Koca et al. reported excellent outcomes in 15 patients following the use of adjuvant yttrium radiosynovectomy after surgical synovectomy. There were no side effects noted, such as leakage of radioactivity, as seen in full-body imaging [12]. Similarly, Shabat et al. found all of the 10 patients treated with adjuvant yttrium intra-articular injection showed no evidence of progression or recurrence of disease [13]. The same results were noted by Kamaleshwaran et al. and Nassar et al. [7,14]. These studies show that yttrium radiosynovectomy is a safe and effective adjuvant treatment after surgery. Karaman et al. noted that patient satisfaction was higher in patients with chronic non-specific recurrent synovitis treated with combined therapy [15]. Although not directly assessing patient satisfaction, this could be inferred from our cohort with caution from the higher KOOS scores in those treated with combined therapy.

Neoadjuvant arthroscopic mechanical debridement is a safe and quick procedure with minimal side effects. It can, in theory, increase the risk of haematoma due to the haemorrhagic nature of the pathology and this may explain the higher pain and initial symptom scores in this group [7].

The side effects of radiosynovectomy can include local reactions such as radio-necrosis extravasation to surrounding tissues, including lymph nodes, and febrile reactions, although none of these reactions were observed in our patients. Local radiosynovectomy also avoids external beam radiation therapy and the need for a total synovectomy.

Other measurements of response to treatment include measuring synovial thickening on MRI; however, this is largely dependent on the aggressiveness of the operating surgeon performing the mechanical debridement, is not uniform for all patients, and is largely subjective to the interpreting radiologist [13].

Finally, although PVNS is considered a benign condition, its effects can be extremely debilitating. If recurrence occurs following treatment, as is sometimes the case, treatment can always be repeated to help preserve the joint and delay the need for major joint replacement surgery.

Limitations of the study, as alluded to previously, include the small sample size of patients for this case series. Although the results are generally positive, these would be difficult to extend to the wider population based on these results alone. The low incidence of PVNS, however, means that greater sample sizes would be difficult to come by in a single-centre study. A further limitation of the study is the follow-up period. A longer-term follow-up period for the study would establish a greater understanding of the frequency of recurrence following combined therapy. This could be augmented by assessing recurrence at approximately 18-24 months post procedure with an MRI. The imaging could be reported by two separate radiologists to attempt to mitigate for the subjectivity found to be an issue with scans initially reported measuring response to treatment.

The results, along with the current established literature, have led to changes within the department to offer greater availability to clinicians and a pathway to offer combined therapy to patients. This may in turn lead to further opportunities to generate research, especially to assess longer-term outcomes in PVNS patients. Another aspect to consider with future research is the symptom profile associated with the condition and subsequent treatment. With the results establishing a higher pain profile in those treated with combined therapy, assessing pre-operative analgesia requirements with post-operative requirements will give a further understanding of response to treatment and QoL.

## Conclusions

From our case series, we have found that the use of combined therapy demonstrated a better short-term outcome compared to radiosynovectomy alone, with regards to KOOS scores. We conclude that neoadjuvant arthroscopic mechanical debridement is a safe and effective treatment with minimal side effects. Following this, a study looking at the long-term comparison of outcomes between the two treatment modalities can be used to help establish best practices in the management of PVNS of the knee.
